# Effect of multiple hook heights and positions during en masse maxillary distalization using infrazygomatic crest miniscrew– single and double points of force application: a finite element analysis study

**DOI:** 10.1186/s12903-025-06138-4

**Published:** 2025-05-23

**Authors:** Maxim Fares, Wessam Marzouk, Hanan A. Ismail, Yasser Abuouf, Hassan E. Kassem

**Affiliations:** 1 Department of Orthodontics, Faculty of Dentistry, Alexandria University, Champollion Street, Azarita, Alexandria, Egypt; 2 Department of Orthodontics, Pharos University, Alexandria, Egypt; 3 Department of Mechanical Engineering, Alexandria University, Alexandria, Egypt

**Keywords:** Infrazygomatic miniscrews, Total distalization, Class II malocclusion, Hook length and position

## Abstract

**Background:**

This study aimed to simulate maxillary dentition distalization as one unit anchored to infrazygomatic crest (IZC) miniscrew using different hook positions and lengths.

**Materials and methods:**

Eleven finite-element models (FEM) were constructed from a cone beam computed tomography scan of a patient with Class II malocclusion. Different force vectors to the IZC miniscrew were simulated using one point of force application either mesial to the canine or mesial to the first premolar, using different hook lengths (0, 2, 4, and 6 mm). In the novel approach, two point-force system was constructed using double-hook retraction in three conditions. The FEM yielded tooth displacement patterns and stress contour plots of the periodontal ligament.

**Results:**

When hooks were placed mesial to the canine, the incisor showed palatal translation with controlled palatal tipping at 0 and 2 mm, palatal bodily displacement at 4 mm, and palatal translation with torquing at 6 mm. In hooks mesial to the first premolar, the pattern showed palatal translation with torquing, except with the 0-mm hook where controlled palatal tipping occurred. Whereas, vertically, it shows extrusion at the 0- and 2-mm hooks mesial to the first premolar and intrusion with the remaining single hook simulations. The molar exhibited translation with controlled distal tipping at all hook lengths mesial to the canine and 0 mm mesial to the first premolar, while it demonstrated distal translation with torquing at 2-,4-, and 6-mm hooks mesial to the first premolar. Vertically, it showed extrusion with hooks mesial to the canine, which changed to intrusion with hooks mesial to the first premolar. In double-hook simulations, the incisor showed bodily displacement only with hooks mesial to the canine and second premolar, whereas the molar showed distal bodily movement with hooks mesial to the first and second premolars.

**Conclusion:**

Hook height and position variations are crucial in the resultant displacement pattern. Accordingly, different force systems should be tailored individually based on the patient’s initial malocclusion.

**Supplementary Information:**

The online version contains supplementary material available at 10.1186/s12903-025-06138-4.

## Background

Class II malocclusion is one of the most prevalent types of malocclusions dealt with daily in orthodontic practice [1]. Depending on the extent of the skeletal and dentoalveolar components of this malocclusion, a broad spectrum of treatment modalities exists, and one of the classical techniques is total distalization of the maxillary dentition [2]. Recent advances in temporary skeletal anchorage devices (TADs) such as miniscrews and miniplates have been suggested as a favorable modality for maxillary distalization taking advantage of absolute anchorage and non-reliance on patient compliance [3]. Moreover, compared with miniplates, miniscrews offer a more attractive option owing to the simplicity of their placement, enabling the orthodontist to perform the procedure and avoiding the surgical complications associated with miniplates.

Two-stage maxillary dentition distalization in patients with non-extraction class II malocclusion using TAD-supported appliances was formerly reported in literature [4]. However, en masse maxillary distalization was made easier with the introduction of extra-alveolar miniscrew insertion sites [5]. The zygomatic buttress and maxillary tuberosity are two sites recommended by many clinicians, as they allow safe insertion of the miniscrew with less possibility of root injury [6].

Different experimental methods have been utilized to simulate multiple force systems and analyze the resultant displacement patterns [7, 8]. One of these powerful tools is finite-element analysis (FEA), which gained its popularity because of its capacity to achieve quantitative visualization of an object in three dimensions [9, 10]. Moreover, it allows simple and objective manipulation of geometrical configurations, material properties, loading conditions, and boundary conditions. Although most of the current literature uses generic models or 3D scan data of virtually constructed models, the simultaneous increase in computing power and software complexity have allowed researchers to make use of 3D radiographs such as cone-beam computed tomography (CBCT) in FEA. Thereby, accurate sculpturing of anatomical models by segmenting the teeth, bone structure, and periodontal ligament (PDL) has been made possible [11].

With FEA simulations, multiple force systems have been proposed for en masse distalization of the maxillary dentition [7, 12, 13]. These systems differ in the position and number of force application points between the teeth, the use of interdental hooks, and their heights. To the best of the authors’ knowledge, three studies have employed the FEA method to predict the appropriate hook length and retraction point with en masse maxillary distalization on an IZC miniscrew [14–16]. However, no consensus has been made in the literature on the appropriate force system to treat class II malocclusion.

The use of FEA to solve intricate biomechanical scenarios has paved the path to explore what appeared to be clinically trivial changes, such as hook lengths and positions. Thus, multiple retraction sites with variable hook lengths and positions were tested in this study using a single IZC site between the maxillary first and second molars, aiming to derive initial tooth displacement patterns associated with different force directions and the resultant stress distributions in the PDL.

## Materials and methods

For the selection of an appropriate CBCT scan, the following criteria were prioritized: a complete set of maxillary permanent dentition, extracted or congenitally missing maxillary third molars, and a clearly visible zygomatic buttress to be included in model designing for an accurate positioning of the IZC miniscrew [17, 18]. CBCT scan of a patient with Class II malocclusion was obtained from the archives of the Orthodontic Department, Faculty of Dentistry, Alexandria University, registered with large field of view (FOV), isotropic voxel size of 75-microns, 8 mA and 90 KVp (Carestream CS 8200, New York, USA) [11]. The study protocol was approved by the institutional review board of the ethical committee at the Faculty of Dentistry, Alexandria University (IRB:00010559).

Digital imaging and communication in medicine (DICOM) file from the CBCT scan was imported to 3D segmentation and modeling software (Mimics 10.02; Materialise, Leuven, Belgium). Such method allowed for the definite representation of different anatomical structures such as the different root morphologies, level of the alveolar bone crest, and zygomatic buttress needed for accurate screw positioning. Then, DICOM data were reconstructed and processed into a standard tessellation language file using 3-Matic (3-matic 10.02; Materialise, Leuven, Belgium), and data were imported into FEA software (Ansys 19.1; Inc., Canonsburg, PA) [11].

### Model construction

**Fig. 1:**
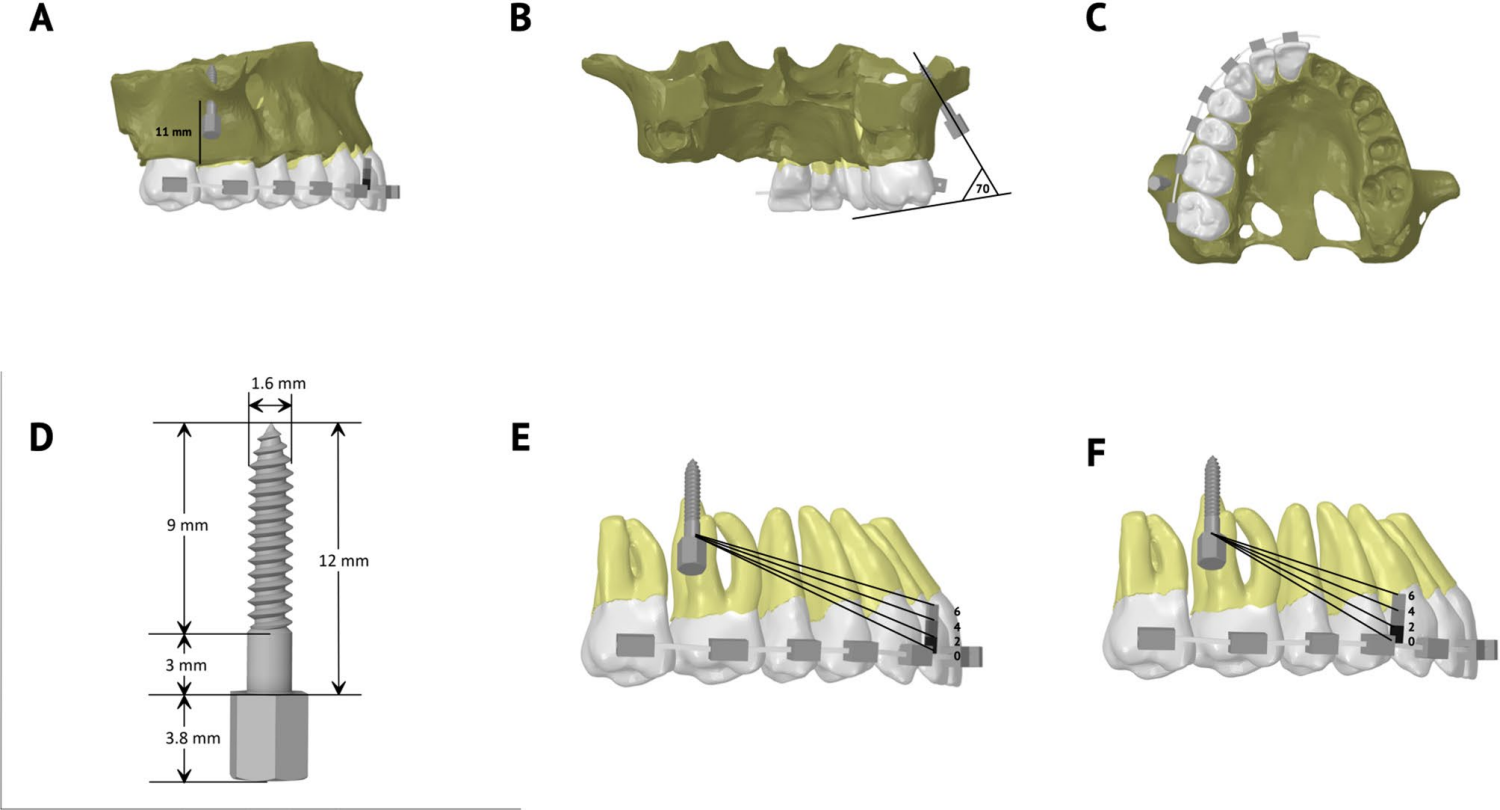
(**A–C**) Illustrations of the 3D structured model with the positioning of the infrazygomatic miniscrew (IZC) in the zygomatic buttress between the first and second molars, 11 mm from the alveolar bone crest and at an insertion angle 70° from the posterior occlusal plane. (**D**) Structure of the IZC miniscrew with the exact length of the threads and the smooth collar. (**E**) Setup of force vectors mesial to the maxillary canine with 0-, 2-, 4-, and 6-mm hook lengths. (**F**) Setup of force vectors mesial to the maxillary first premolar with 0-, 2-, 4-, and 6-mm hook heights

Assuming bilaterally symmetric boundary conditions of the dentition, only the right side of the arch was modeled and processed in the FEA software (Fig. 1A) [7]. It was then oriented such that the occlusal plane coincides with the Y-axis, and the median palatine raphe is perpendicular to the x-axis to facilitate the processing of results in the 3 coordinates. Young’s modulus and Poisson’s ratio for different model components are provided in Table 1. Each tooth was divided into shell elements and defined as a rigid body without differentiation into enamel and dentin [13]. Contact elements were also overlaid on adjoining surfaces of the coronal structure and defined as bonded. The PDL, assumed to be a linear elastic film, was constructed to have a uniform thickness of 0.2 mm as an overlying structure to the root surface [7]. Moreover, the alveolar bone was assumed to be a rigid body. Because its Young’s modulus was significantly larger than that of the PDL, it was structured only for accurate positioning of the IZC miniscrews (Fig. 1A and C) [19]. Because the outer surface of the PDL is attached to bone, the nodes of the external surface of PDL were selected and assumed to be fixed as a substitute for meshing of the entire bone [7].

**Table 1:** Material properties

Component	Young’s Modulus (MPa)	Poisson’s ratio
Teeth	20,000	0.3
Bone	2000	0.3
PDL*	0.2	0.4
Stainless steel	200,000	0.3

* PDL: Periodontal ligament

The archwire was constructed from 0.019 × 0.025-in stainless-steel wire and divided into 3D solid elements [20]. 3D symmetrical boundary conditions were applied at the median end of the archwire to constrain the finite-element model (FEM) [7]. Brackets were constructed with a 0.022 × 0.028-inch slot, divided into shell elements, and defined as rigid bodies. Contact elements were overlaid between the archwire and brackets and defined as frictional contact, with a frictional coefficient of 0.15 [7, 13]. Moreover, the angle of play between the archwire and bracket slot was designed such that the wire was parallel to the bracket wall with a uniform clearance gap before force application. The interface between the teeth and brackets was considered completely joined to eliminate the effect of the bonding material [13].

### IZC Miniscrew design and positioning

IZC miniscrew was modeled using SolidWorks (Dassault Systemes Americas, Waltham, MA, USA) (Fig. 1D). Accurate positioning of the IZC miniscrew between the maxillary first and second molars was achieved using the segmented zygomatic buttress, 11 mm apical to the alveolar crest and at 70-degree angulation from the posterior occlusal plane (Fig. 1A and C) [5, 18, 19, 21].

### Force vector simulations

Two factors were investigated in this study: the effect of changing the height of the retraction hook on the distalization pattern and stress distribution, and the effect of different interdental points of force application. In each simulation, a force of 4 N was applied to different hook positions and lengths [15]. The analysis of force into three components (X, Y, and Z) was performed using mathematical equations to ensure the accurate 3D application of force system from the screw neck to the appropriate hook length.

### Design of FEM simulations

**Fig. 2:**
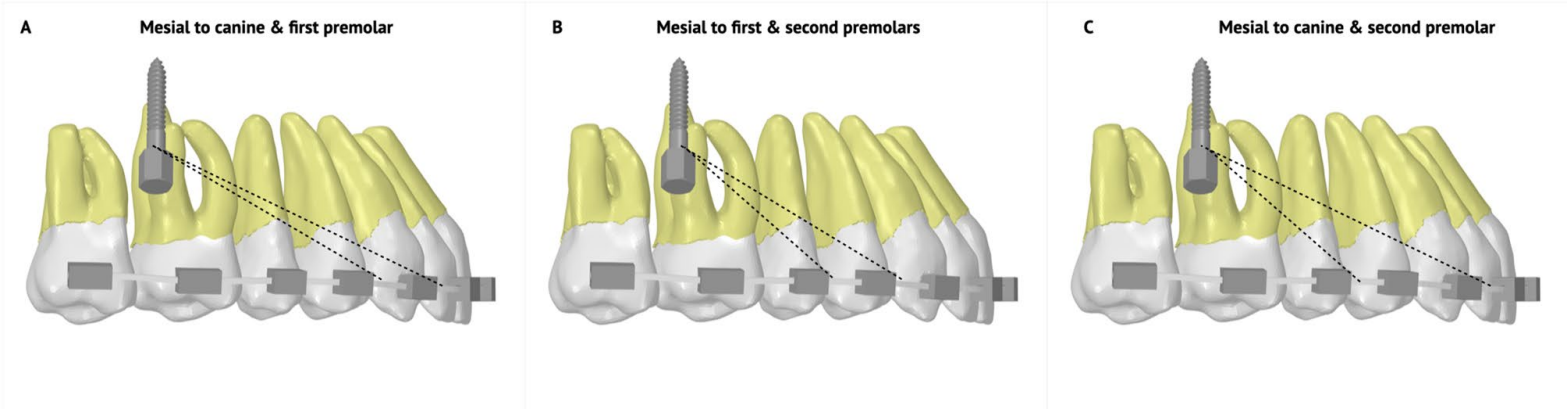
A representation of the direction of force vectors in double-hook simulations. (**A**) Archwire-level double hooks mesial to the canine and first premolar. (**B**) Archwire-level double hooks mesial to the first and second premolars. (**C**) Archwire-level double hooks mesial to the canine and second premolar

Eleven FEMs were constructed; 4 models with single hook mesial to the maxillary canine, and 4 models with single hook mesial to the maxillary first premolar, each with 4 different hook heights (0-, 2-, 4- and 6-mm) (Fig. 1E and F). Three of the aforementioned FEMs were double-hook simulations and were designed with archwire-level hooks mesial to the canine and first premolar; mesial to the canine and second premolar; and lastly mesial to the first and second premolars (Fig. 2).

### Definition of coordinate systems

A standard global coordinate system was set for the entire FEM and defined as follows: The X-axis corresponds to the mesiodistal direction for the incisors and buccopalatal for the canines and posterior teeth, the Y-axis corresponds to the anteroposterior direction, and the Z-axis corresponds to the superoinferior direction. A + x value was defined as medial direction for the anterior teeth and palatal direction of the posterior teeth, +y as posterior direction, and + z as apical (inferior) direction.

### Analysis of tooth displacement and stress distribution

**Fig. 3:**
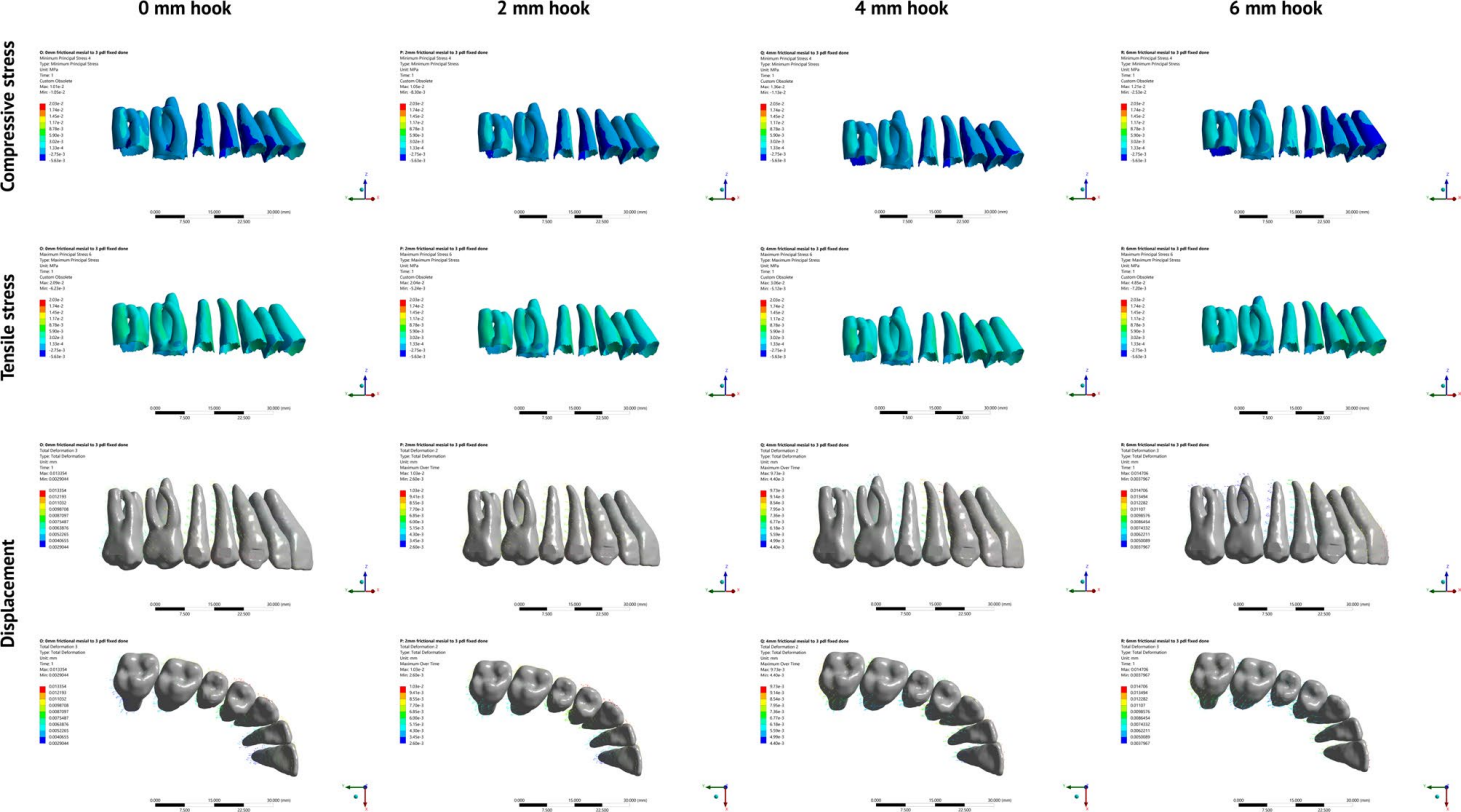
Displacement vectors and stress contour plots representing the movement directions and stress distribution during total maxillary distalization using multiple hook lengths (0, 2, 4, and 6 mm) with single hook mesial to the maxillary canine on an IZC miniscrew

**Fig. 4:**
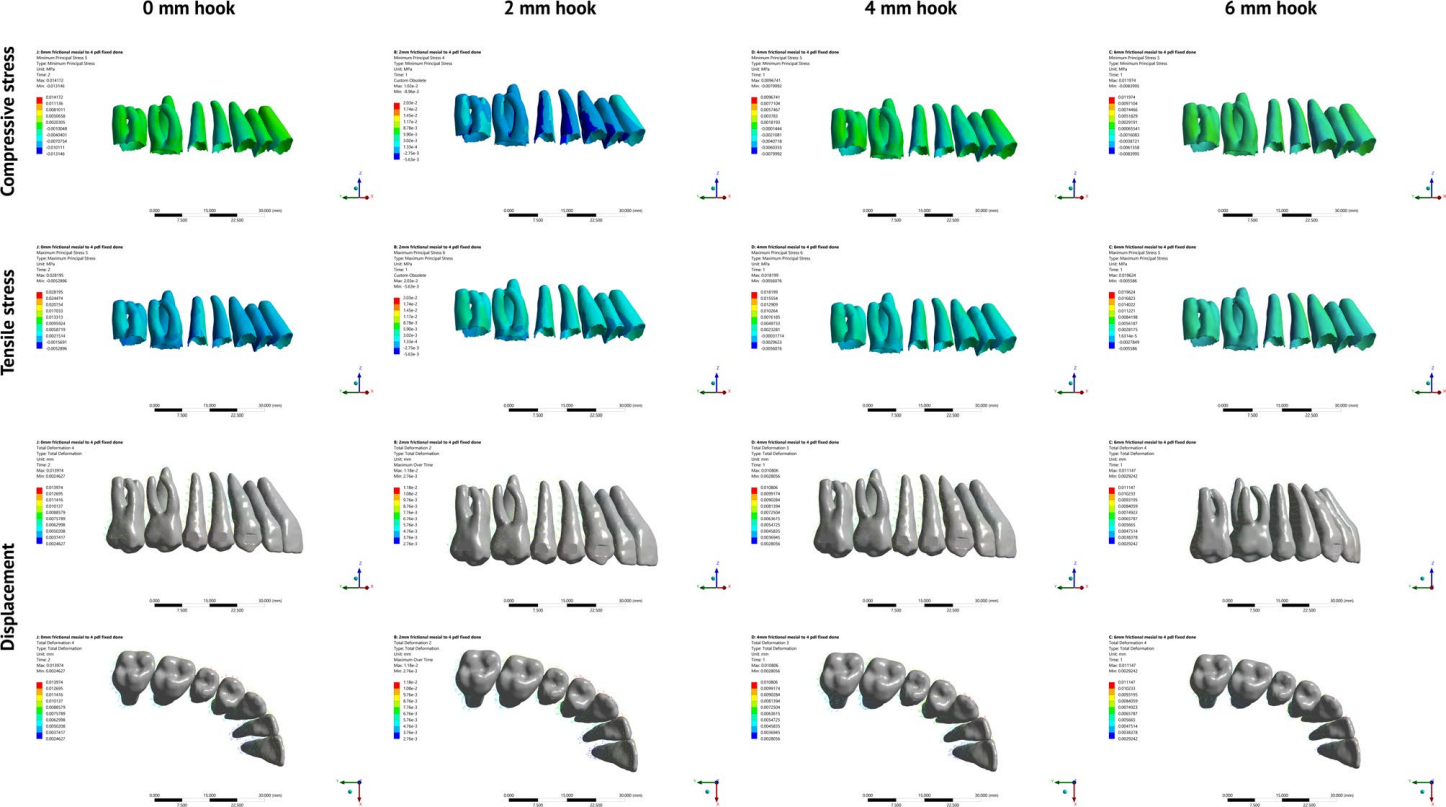
Displacement vectors and stress contour plots representing the movement directions and stress distribution during total maxillary distalization using multiple hook lengths (0, 2, 4, and 6 mm) with single hook mesial to the maxillary first premolar on an IZC miniscrew

**Fig. 5:**
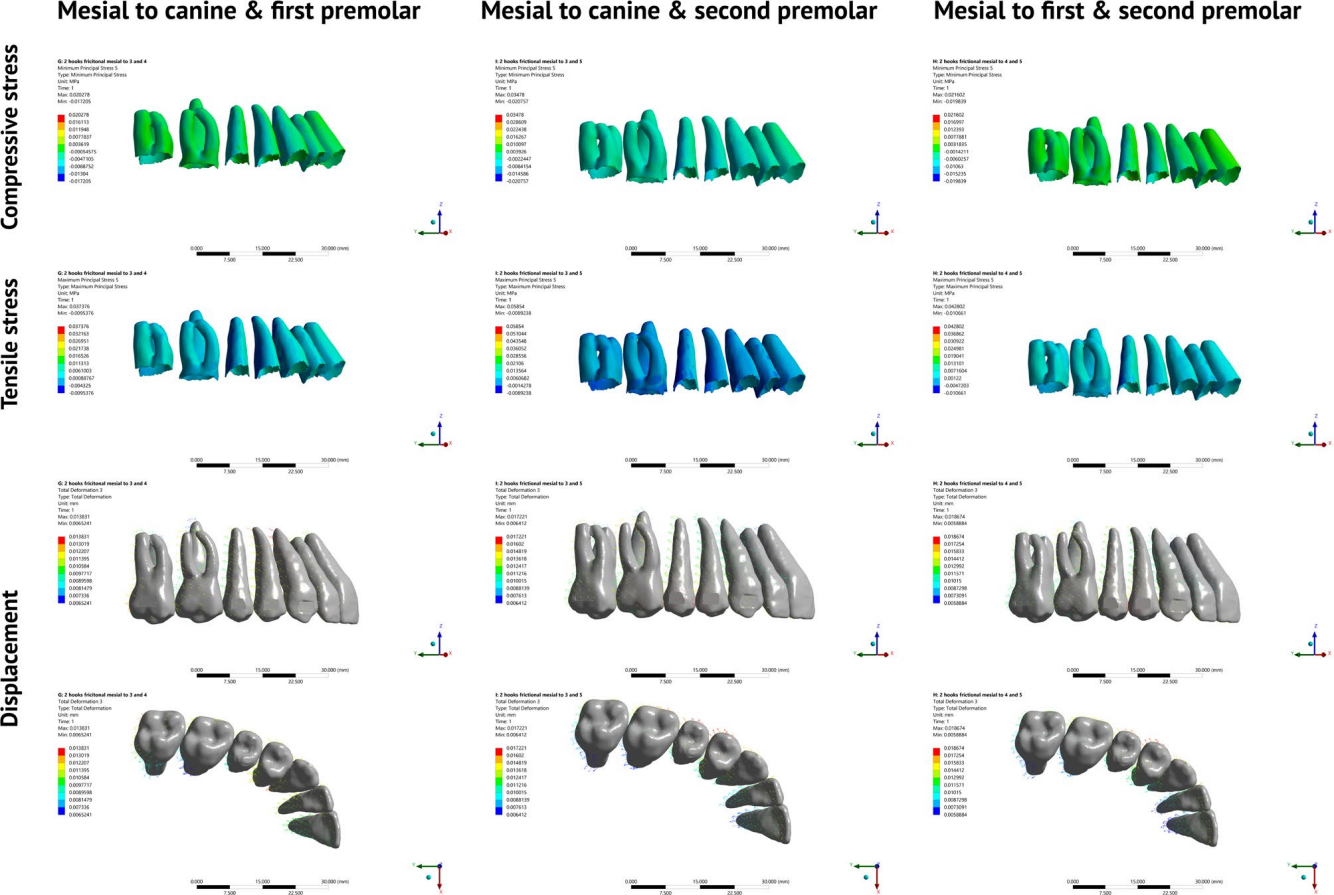
Displacement vectors and stress contour plots representing the movement directions and stress distribution during total maxillary distalization using double-archwire hook retraction on an IZC miniscrew

Displacement and stress distribution patterns were examined for three teeth in each FEM: maxillary central incisor, maxillary canine and maxillary first molar. Two landmarks were determined for each tooth (Figure S1): one at the midpoint of the incisal edge of the central incisor, cusp tip of the canine, and mesiobuccal cusp tip of the first molar, and another on the root apex of the incisor, canine and mesiobuccal root of the first molar. Displacement of the crown and root points was measured relative to the global coordinate system for each tooth in three planes (X, Y, and Z) [22]. Buccolingual rotation of the incisor, and buccolingual and mesiodistal rotations of the canine and first molar were calculated from the coordinates of the crown and root points for each tooth using mathematical Eq. (7). Maximum and minimum principal stresses were analyzed, and the maximum values for each tooth were recorded and used to derive compressive and tensile stress contour plots Figs. 3 and 4, and 5. The crown coordinates of the maxillary central incisor and first molar were used to determine the corono-apical rotation of the occlusal plane relative to the X-axis.

## Results

Displacement and stress distribution for single hook simulations mesial to canine and first premolar are represented in Figs. 3 and 4 respectively. Whereas for double hook simulations, displacement and stress distribution are represented in Fig. 5.

### Single-hook simulations

#### Incisors

Regarding incisor displacement with hooks mesial to the maxillary canine in Table 2, palatal displacement was associated with negative root torque with an amount that decreased gradually as the hook length increased and changed to positive torque at 6-mm hook (Fig. 3). Vertically, intrusive movement occurred and increased with the hook height.

**Table 2:** Three-dimensional displacement of the maxillary teeth with hooks mesial to maxillary canine

Tooth	Direction	Measurement point	Displacement (x 10^− 2^ mm) Hook length (in mm)			
			0	2	4	6
Incisor	Antero-posterior	Crown	1.363	0.932	0.609	0.335
		Root	0.408	0.592	0.508	0.629
	Mesio-distal	Crown	1.846	1.977	1.553	1.551
		Root	-0.151	-0.384	-0.494	-0.768
	Occluso-cervical	Crown	0.68	1.155	1.062	1.345
		Root	1.013	1.176	0.988	1.067
Canine	Antero-posterior	Crown	0.898	0.922	0.979	0.997
		Root	0.374	0.434	0.438	0.496
	Labio-palatal	Crown	1.586	1.843	1.556	1.602
		Root	-0.309	-0.528	-0.519	-0.6
	Occluso-cervical	Crown	-0.136	-0.231	-0.343	-0.413
		Root	0.414	0.387	0.245	0.181
Molar	Antero-posterior	Crown	0.471	0.511	0.661	0.722
		Root	0.123	0.033	0.067	0.026
	Bucco-palatal	Crown	0.462	0.683	0.667	0.777
		Root	-0.043	-0.014	-0.026	-0.029
	Occluso-cervical	Crown	-0.07	-0.232	-0.26	-0.364
		Root	0.027	-0.097	-0.103	-0.181

In hooks mesial to the maxillary first premolar (Table 3), palatal displacement with negative root torque occurred only at the 0-mm hook, which changed to positive torque with the 2-mm hook and progressively increased as the hook height increased (Fig. 4). Vertically, extrusive movements were shown at 0-mm, which decreased gradually and changed to intrusion with the 4-mm hook.

**Table 3:** Three-dimensional displacement of the maxillary teeth with hooks mesial to 1st premolar

Tooth	Direction	Measurement point	Displacement (x 10^− 2^ nm) Hook length (in mm)			
			0	2	4	6
Incisor	Antero-posterior	Crown	0.341	0.183	0.08	0.013
		Root	0.186	0.298	0.381	0.417
	Mesio-distal	Crown	-0.425	-1.014	-0.946	-0.651
		Root	0.367	0.295	0.234	0.183
	Occluso-cervical	Crown	-0.153	-0.064	0.141	0.246
		Root	-0.105	-0.041	0.068	0.123
Canine	Antero-posterior	Crown	0.314	0.238	0.199	0.173
		Root	0.412	0.61	0.773	0.919
	Labio-palatal	Crown	-0.398	-0.925	-0.804	-0.53
		Root	0.433	0.277	0.147	0.036
	Occluso-cervical	Crown	0.613	0.649	0.666	0.671
		Root	0.314	0.282	0.259	0.219
Molar	Antero-posterior	Crown	0.47	0.5	0.558	0.648
		Root	0.808	0.752	0.689	0.386
	Bucco-palatal	Crown	-0.254	-0.491	-0.298	-0.131
		Root	0.125	0.014	-0.064	-0.108
	Occluso-cervical	Crown	0.825	0.585	0.382	0.212
		Root	0.709	0.505	0.342	0.135

#### Canines

The canine showed a gradual increase in distal tipping and extrusive movement as the hook length increased in the case of hooks mesial to it. In hooks mesial to the first premolar, distal tipping and intrusion were found at all hook lengths.

#### Molars

In hooks mesial to the canine, the molar showed distal tipping and extrusion at the archwire-level retraction that increased gradually as hook length increased (Fig. 3). The increased negative crown inclination is demonstrated in Table 4. In hooks mesial to the first premolar, the magnitude of distal displacement progressively increased with decreased intrusive movement as the hook length increased (Table 3**).** Table 4 shows that the changes in the torque were positive at all hook lengths, with minimal inclination change with the 6-mm hook.

**Table 4:** Buccopalatal and mesiodistal rotations for single Hook simulations

Tooth	Hook position	Rotations (x 10^− 2^ degree) Hook length (in mm)			
		0	2	4	6
Incisor Bucco-palatal	Hook mesial to canine	-2.534	-0.782	-0.135	0.995
	Hook mesial to 1st premolar	-0.404	0.228	0.759	1.048
Canine Mesio-distal	Hook mesial to canine	-1.763	-1.751	-1.841	-1.757
	Hook mesial to 1st premolar	0.531	1.228	1.732	2.175
Canine Bucco-palatal	Hook mesial to canine	5.313	6.616	5.812	6.152
	Hook mesial to 1st premolar	-2.356	-3.38	-2.728	-1.722
Molar Mesio-distal	Hook mesial to canine	-1.032	-1.418	-1.759	-2.059
	Hook mesial to 1st premolar	1.014	0.752	0.388	-0.7
Molar Bucco-palatal	Hook mesial to canine	1.485	2.052	2.041	2.373
	Hook mesial to 1st premolar	-1.121	-1.484	-0.687	-0.077

### Double-hook simulations

#### Incisors

Both the crown and root points showed palatal movement in the three conditions, as shown in Table 5**and** Fig. 5. Vertically, both the crown and root points showed intrusive movement in hooks mesial to the canine and first premolar. With hooks mesial to the first and second premolars, the crown point extruded. The incisor showed palatal bodily displacement with negligible vertical movement in hooks mesial to the canine and second premolar. Positive root torque occurred with hooks mesial to the canine and first premolar, and changed to negative torque with hooks mesial to the first and second premolars (Table 6).

**Table 5:** Three-dimensional displacement of the maxillary teeth with double hooks

Tooth	Direction	Measurement point	Displacement (x 10^− 2^ nm) Hook position		
			Mesial to canine & 1st premolar	Mesial to 1st & 2nd premolars	Mesial to canine & 2nd premolar
Incisor	Antero-posterior	Crown	0.721	0.765	0.737
		Root	0.984	0.531	0.734
	Mesio-distal	Crown	-0.691	-1.21	-1.029
		Root	0.129	0.457	0.257
	Occluso-cervical	Crown	0.463	-0.206	-0.062
		Root	0.384	0.036	0.04
Canine	Antero-posterior	Crown	0.581	0.525	0.58
		Root	1.262	0.922	0.949
	Labio-palatal	Crown	-0.638	-1.239	-0.967
		Root	-0.219	0.466	0.079
	Occluso-cervical	Crown	0.844	0.996	0.841
		Root	0.474	0.508	0.457
Molar	Antero-posterior	Crown	0.941	0.769	0.83
		Root	0.776	0.768	0.782
	Bucco-palatal	Crown	-0.388	-0.892	-0.672
		Root	-0.134	-0.336	-0.252
	Occluso-cervical	Crown	0.519	0.999	0.939
		Root	0.496	0.752	0.713

**Table 6:** Mesiodistal and buccopalatal rotations for double Hook simulation

Tooth	Rotations (x 10^− 2^ degree) Hook position		
	Mesial to canine & 1st premolar	Mesial to 1st & 2nd premolars	Mesial to canine & 2nd premolar
Incisor Bucco-palatal	0.682	-0.818	-0.134
Canine Mesio-distal	1.941	1.4102	1.239
Canine Bucco-palatal	-1.291	-4.778	-2.971
Molar Mesio-distal	-0.454	0.123	-0.017
Molar Bucco-palatal	-0.742	-1.649	-1.25

#### Canines

Distal movement occurred at both the crown and root points with a greater amount apically than coronally. Vertically, the crown and root points showed intrusive movements, with the magnitude of intrusion at the crown point greater than that at the root point in all cases. Positive root torque occurred in all conditions (Table 6).

#### Molars

Distal movement occurred at both the crown and root points in all conditions. With hooks mesial to the canine and first premolar, the magnitude of distal displacement at the crown point was more than that at the root, resulting in a distal crown tip. With hooks mesial to the first and second premolars, the molar showed distal bodily translation. Intrusive movements occurred at the crown and root points in all cases. Table 6 shows that the torquing pattern of the molar is opposite to that of the incisor.

### Stress distribution

#### Single-hook simulations

In hooks mesial to the canine, the incisor showed the least value of compressive stresses with 4-mm hooks (Table 7; Fig. 3). As for the molars, compressive stress increased gradually as the hook length increased. In hooks mesial to first premolar, the incisors and molars showed a comparable pattern with the maximum compressive stress with the 0-mm hook, which tended to decrease gradually as the hook length increased to reach the minimum value with the 4-mm hook for the incisor and 6-mm hook for the molar where bodily translation occurred (Fig. 4).

**Table 7:** Maximum compressive & tensile stresses on periodontal ligament (x 10− 2 MPa)

Hook position	Hook length (mm)	Central incisor		Canine		Molar	
		Compressive	Tensile	Compressive	Tensile	Compressive	Tensile
Mesial to canine	0	3.518	2.229	1.191	0.889	0.374	0.436
	2	4.453	1.396	1.271	1.068	0.445	0.34
	4	3.003	1.457	1.161	1.234	0.518	0.252
	6	3.77	1.871	1.18	1.364	0.57	0.164
Mesial to 1st premolar	0	1.016	8.064	0.6889	1.621	0.644	0.088
	2	0.615	9.033	0.772	1.674	0.585	0.075
	4	0.432	5.574	0.786	1.555	0.477	0.095
	6	0.484	6.136	0.820	1.626	0.458	0.098
Mesial to canine & 1st premolar	0	1.02	2.969	1.369	2.428	0.8	0.501
Mesial to canine & 2nd premolar	0	1.599	5.854	1.216	1.966	1.067	1.051
Mesial to 1st & 2nd premolars	0	1.54	2.977	1.146	1.929	1.223	0.945

#### Double-hook simulations

For the incisors, the highest maximum compressive stress occurred with hooks mesial to the canine and second premolar, whereas the lowest stress value occurred with hooks mesial to the canine and first premolar (Table 7; Fig. 5). Similarly, the molar showed the least stress with hooks mesial to the canine and first premolar; however, the highest compressive stress took place with hooks mesial to the first and second premolars.

### Occlusal plane rotation

#### Single-hook simulations

The occlusal plane showed increased counterclockwise (flattening) rotation as the hook length increased mesial to the canine. While with hooks mesial to the first premolar, the occlusal plane showed clockwise (steepening) rotation in the case of archwire-level retraction, which decreased gradually and changed to counterclockwise rotation with the 6-mm hook (Table 8**).**

**Table 8:** Rotation of the occlusal plane for single and double Hook simulations

Hook position	Rotations (x 10^− 2^degree) Hook length (in mm)			
	0	2	4	6
Mesial to canine	1.532	2.997	2.917	3.811
Mesial to 1st premolar	-2.135	-1.387	-0.466	0.1602
Mesial to canine & 1st premolar	-0.993			
Mesial to 1st & 2nd premolars	-0.291			
Mesial to canine & 2nd premolar	-0.168			

#### Double-hook simulations

Table 8 shows that clockwise (steepening) rotation of the occlusal plane occurred in all double-hook conditions.

## Discussion

The treatment of patients with Class II malocclusion has always been a subject of interest in modern orthodontics [2, 3]. Recent advances in TADs, particularly extra-alveolar miniscrews, have led the way to total arch distalization as a substitute for extraction techniques [5, 8, 15]. Recent literature suggested that the position and height of the retraction hooks used in conjunction with the IZC may have affected the resultant force system [5]. Accordingly, varying the hook position and height may be tailored to the clinical presentation of every case. Therefore, this study focused on the FEA method to test different force applications using variable hook positions and lengths and their effect on the amount and direction of resultant tooth displacement and on PDL stress distribution.

The anteroposterior molar displacement with variable hook lengths mesial to the canine showed distal translation with controlled distal tipping. With hooks mesial to the maxillary first premolar, distal translation with torquing pattern was noted, except for the 6-mm hook that showed a pattern consistent with hooks mesial to the canine. An interpretation could be reached by analyzing the force components in the X, Y, and Z coordinates, where the anteroposterior force vector in the Y-axis increased gradually from 0 to 6 mm hook length mesial to the canine, which is consistent with the gradual increase in the magnitude of distal tipping. On the contrary, with the hooks mesial to the first premolar, given the pronounced vertical component of force in the Z coordinate, distal translation with a torquing pattern was noted. However, the magnitude of torquing decreased gradually as the hook height increased till reaching the 6-mm hook length, where the pattern changed to controlled distal tipping.

Vertically, the molar showed extrusion with a progressively increasing pattern with hooks mesial to the maxillary canine. This is consistent with the increased magnitude of distal tipping as the hook length increased. In addition, the influence of the anterior point of force application could be relatively explained by the point of dissociation and its effect on the moment to force equilibrium while considering the rigidity and length of the archwire, which in turn could contribute to the extrusive vertical pattern of the molars [23]. Conversely, the molar showed intrusion with hooks mesial to the first premolar, possibly due to the pronounced vertical component of force as the point of force application moved posteriorly [13].

Analyzing the horizontal component of force in the X-coordinate and its effect on the palatal twisting tendency of the archwire might contribute significantly to our understanding of force vector and its relationship with the resultant tooth movement of the molars. With hooks mesial to the maxillary canine, the moment generated from archwire torsion was greater than that with hooks mesial to the first premolar. As a result, such moments overcome those generated from the force relative to the center of resistance of the arch, causing palatal tipping of the molars. The opposite occurred with hooks mesial to the first premolar, where the moment of force was higher than that of archwire twisting, thus buccal tipping occurred. As the hook length increased mesial to the first premolar, the effect of archwire torsion became more pronounced and is manifested with the 6-mm hook by balancing the buccal tipping moment, thus achieving molar bodily displacement. These findings agree with the results of Wu et al. [12] in the horizontal direction, where the molar showed palatal tipping movement with the 4-mm hook mesial to the canine.

Independent of the point of force application, the anterior segment exhibited major changes compared with the molars concerning the displacement patterns and stress distribution, regardless of the hook length [16, 24]. One of the possible explanations for the variation in the anteroposterior displacement trend of the incisor is the balance between the moment generated from the force vector relative to the center of resistance of the incisor and the moment produced from archwire deflection. At 0- and 2-mm hooks mesial to the canine and 0-mm hook mesial to the first premolar, the moment generated from the force vector was greater, causing a tipping pattern. At 4-mm hook mesial to the canine, equilibrium is reached, causing both the crown and root to move nearly equally in the same direction. In the remaining conditions, the moment from archwire deflection predominated, leading to torquing movement.

Vertically, the incisors showed progressively increasing intrusive movement as the hook length increased, with hooks mesial to the maxillary canine. Such a pattern could also be attributed to the moment generated from the archwire deflection, causing an overpowering effect on the moment of the force system relative to the center of resistance of the incisor, owing to the higher force magnitude applied [25]. With hooks mesial to the first premolar, the pattern for the incisors was consistent with the direction of force vector relative to the center of resistance of the entire arch, where extrusion occurred with 0- and 2-mm hooks passing below the center of resistance and then reverted to intrusion with 4- and 6-mm hooks. Generally, the magnitude of coronal displacement anteroposteriorly and buccopalatally in hooks mesial to the first premolar was lower than that mesial to the canine, independent of the hook height, which reflects the influence of the shorter lever arm in the former because the point-of-force application is closer to the IZC miniscrew position [13].

These results coincide with what Sanap et al. [14] and Khan et al. [15] reported in their FE studies, except for 0-mm hook height in the latter, where incisor extrusion and molar intrusion occurred. Schwertner et al. [16] also reported extrusion of the incisors at 4- and 7-mm hooks mesial to the canine, but with a force magnitude of 3.4 N, as opposed to 4 N in this study. Wu et al. [12] and Rosa et al. [8], in their clinical trials, reported similar anteroposterior incisor and molar displacement trends, whereas vertically contrary to our findings, the incisor displayed an extrusive displacement pattern while the molar exhibited an intrusive one. This could be explained in terms of the higher force magnitude used in the present study causing an overpowering effect on the archwire rigidity, and as a result, the vertical bowing of the wire leading to incisor intrusion [25]. Additionally, a resilient 0.017 × 0.025-inch TMA wire was used in 0.022-inch bracket slots in the study by Rosa et al., which allowed greater vertical bowing of the archwire [20].

With double-hook simulations, the molar and incisor displacement patterns are nearly consistent with the former explanations of the relationship between the force vector and the effect of archwire deflection and torsion. However, with hooks mesial to first and second premolars, the molar showed bodily distal translation, suggesting that the force vector passed close to or even through the center of resistance of the molar. Conversely, the incisor showed bodily displacement with negligible vertical movement in hooks mesial to the canine and second premolar, possibly due to the moment of the archwire deflection balancing that of the force vector relative to the center of resistance of the incisor; as a result, the crown and root moved simultaneously in the same direction.

Regarding the stress distribution pattern in single-hook simulations, the incisor showed higher values in the case of mesial to canine simulations. This observation aligns with the significant extent of tooth movements noted in this area and the closeness of the incisor and canine to the point of force application. Conversely, the molar showed higher stresses with hooks mesial to the first premolar as the point of force application moved more posteriorly. With double-hook simulations, the PDL stress distribution trend became nearly evenly distributed between the incisor and molar, which may be explained by the multiple points of force application. Such a trend should be carefully interpreted to assist in avoiding or at least reducing the risk of root resorption frequently reported with orthodontic tooth movements [26].

In general, the resultant force systems and occlusal plane rotations in most simulations could not be interpreted relative to the center of resistance of the entire arch; therefore, the displacement trend could not be simply anticipated accordingly. Multiple reasonable explanations exist for such a finding; first, the complexity of the force system and its products extend beyond a simple two-dimensional line of force with the limited possibility of passing above, at, or below a definite virtual point. Second, the incorrect assumption of the rigidity of the entire arch moving as a single unit anchored to an IZC miniscrew. Such an assumption is inaccurate because the effect of archwire elasticity is a well-documented fact that cannot be overlooked, depending on the archwire size and material used [20, 27, 28]. Consequently, the FEA could be considered an appropriate tool for simulating complex orthodontic biomechanical scenarios while considering multiple variables such as archwire diameter, torsion, and force magnitude, rather than a simple representation of force directions relative to the center of resistance.

Because the displacements reported in this study are the initial values within the PDL thickness, the meshing and simulation processing did not incorporate the bone. Instead, the outer PDL surface, serving as a substitute for the bone, was deemed fixed in the analysis [7]. The PDL was considered a linear elastic film because nonlinearity will only affect the stress magnitude, not the actual trend of displacement or stress distribution [13, 29]. The use of rigid stainless-steel archwire of large diameter, which reduces the clearance gap, aimed at achieving displacement mostly by dentition movement and thus reduces the effect of the contact angle on the tipping of the incisors and molars [20]. Liou et al. [30]concluded that a force of 400 g per side could be safely used with an infrazygomatic miniscrew without clinical complications.

En masse maxillary distalization has become quite trending recently, both with fixed appliances and clear aligner therapy. However, given the lack of literature data to guide the clinician for the appropriate force system and mechanics, the FEA offers a fair alternative with enough evidence supporting its applicability and validity. Accordingly, the present findings can be applied to achieve better treatment outcomes tailored to the patient’s individual malocclusion. Nevertheless, FEA simulations are challenged with multiple limitations, as they produce only biomechanical data, disregarding the influence of growth, the effect of occlusion, and individual variations among patients. Consequently, their results should be interpreted carefully to avoid misleading generalizations. To overcome such limitations, clinical trials should be similarly designed and executed to confirm or otherwise invalidate the results.

## Conclusions

1. Bodily retraction of the incisors could only be achieved with double hooks retraction mesial to the canine and second premolar, whereas the remainder of the conditions showed palatal tipping or torquing.
2. The molars showed bodily displacement with double hook simulation mesial to the first and second premolars, whereas distal tipping was the predominant trend in the rest of the conditions.
3. Intrusion of the incisors occurred in all single and double hook conditions except for 0- and 2-mm single hooks mesial to the first premolar and double hooks mesial to the first and second premolars, where extrusion occurred.
4. Molars showed intrusion in all single hook simulations mesial to the first premolar and double hook simulations, whereas extrusion occurred with single hook simulations mesial to the canine.
5. Single hook retraction concentrated the stress at the teeth adjacent to the hook position. In contrast, double-hook retraction offered the advantage of even stress distribution between the anterior and posterior units which may reduce the risk of root resorption associated with IZC screw anchored total maxillary arch displacement.
6. The occlusal plane steepened with all simulations except for single hook simulations mesial to the canine and 6-mm hook simulations mesial to the first premolar, where the occlusal plane flattened.

## Electronic supplementary material

Below is the link to the electronic supplementary material.

Supplementary Figure 1: Two landmarks were determined one at the midpoint of the incisal edge of the incisor, cusp tip of the canine and mesiobuccal cusp tip of the 1st molar (**A**) and another on the root apex (**B**) to aid in the analysis of the FEMs findings

## Data Availability

The datasets used and/or analysed during the current study are available from the corresponding author on reasonable request.

## Abbreviations

IZC: Infrazygomatic crest
FEM: Finite-element model
FEA: Finite-element analysis
CBCT: Cone-beam computed tomography
FOV: Field of View
3D: Three dimensional
PDL: Periodontal ligament
DICOM: Digital imaging and communication in medicine
TAD: Temporary anchorage device
