# Supplementary figures and images for: Effect of multiple hook heights and positions during en masse maxillary distalization using infrazygomatic crest miniscrew– single and double points of force application: a finite element analysis study
Source: BMC Oral Health. 2025 May 23;25:771. doi: 10.1186/s12903-025-06138-4 (PMC12100866; doi:10.1186/s12903-025-06138-4)

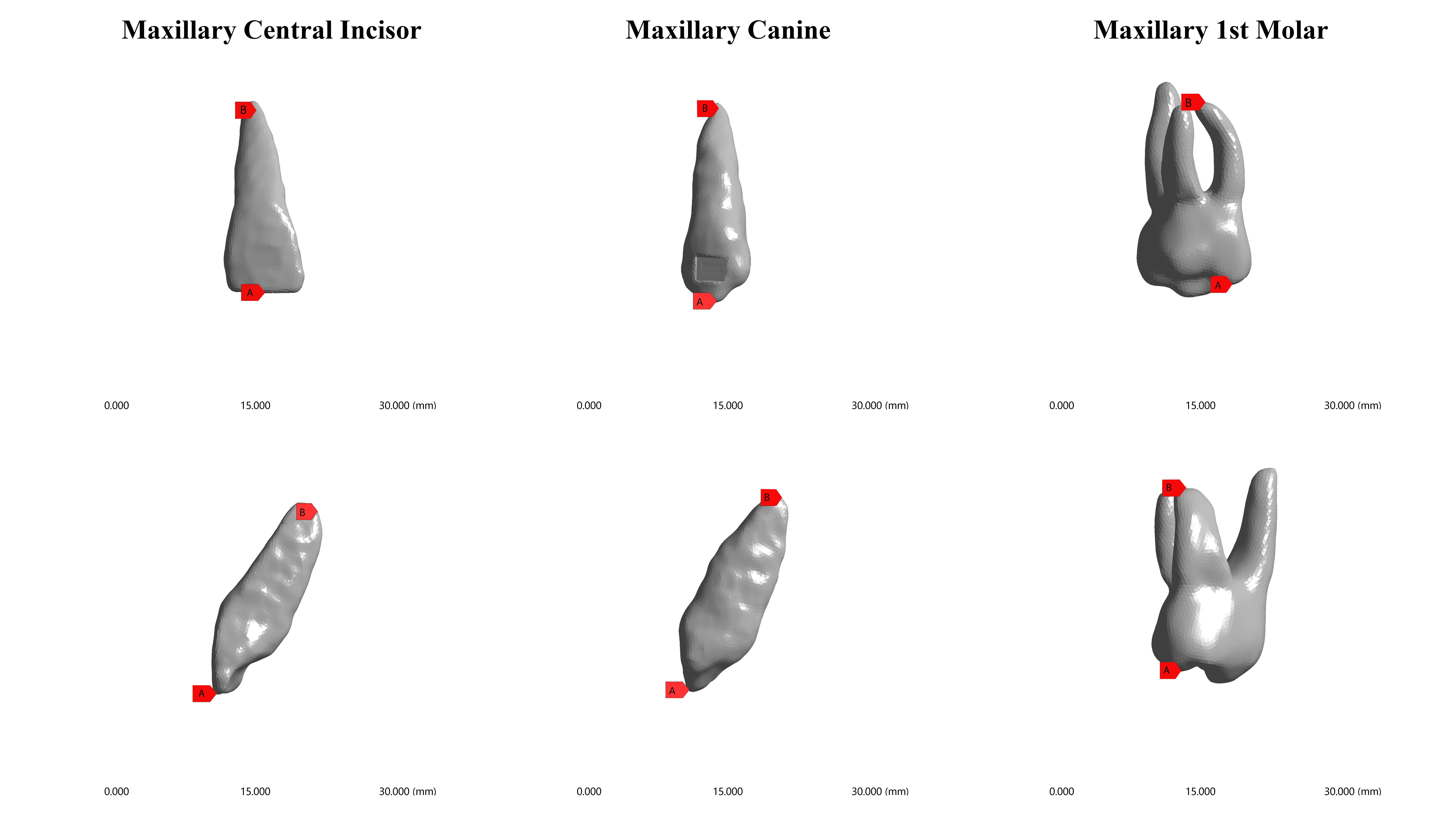

Supplement: Supplementary file 1 — Supplementary Figure 1: Two landmarks were determined one at the midpoint of the incisal edge of the incisor, cusp tip of the canine and mesiobuccal cusp tip of the 1st molar (A) and another on the root apex (B) to aid in the analysis of the FEMs findings [file 12903_2025_6138_MOESM1_ESM.png]
